# Regulatory T cells may participate in *Helicobacter pylori* persistence in gastric MALT lymphoma: lessons from an animal model

**DOI:** 10.18632/oncotarget.6492

**Published:** 2015-12-07

**Authors:** Amandine Marine Laur, Pauline Floch, Lucie Chambonnier, Lucie Benejat, Victoria Korolik, Alban Giese, Pierre Dubus, Francis Mégraud, Antonio Bandeira, Philippe Lehours

**Affiliations:** ^1^ University Bordeaux, Bacteriology Laboratory, Bordeaux, France; ^2^ INSERM U853, Bordeaux, France; ^3^ Institute for Glycomics, Griffith University, Nathan QLD, Australia; ^4^ University Bordeaux, EA 2406, Bordeaux, France; ^5^ Unit for Biology of Lymphocyte Populations, Immunology Department, Institut Pasteur and CIMI, Unity of Treg Biology and Therapy, University of Pierre & Marie Curie, Paris, France

**Keywords:** MALT lymphoma, regulatory T cell, animal model, Helicobacter pylori

## Abstract

It has been postulated that the emergence of autoimmune gastritis in neonatal thymectomised (d3Tx) BALB/c mice may be a consequence of post-surgery deficit in Tregs. In this study, previously obtained samples from d3Tx mice were used in order to determine whether thymectomy creates a deficit in this T cell subset thereby allowing the emergence of autoimmune phenomena as a prerequisite for GML. The splenic Treg reserve and the local recruitment of these cells in the gastric mucosa were investigated using complementary molecular and immunohistochemistry approaches. Higher Foxp3/CD3 ratios were found in the spleen of non-infected d3Tx mice compared to non-thymectomised (NTx) controls. These results indicate a relative enrichment of Tregs following thymectomy in adult mice. The absence of Treg depletion in d3Tx mice is in line with the absence of auto-immune gastritis in non-infected d3Tx mice. Higher levels of T cell and Treg infiltration were also found in the stomach of GML-developing d3Tx mice versus NTx mice. Surprisingly, inflammatory scores inversely correlated with the bacterial inoculum. The presence of a small Treg containing compartment among gastric biopsies of GML developing d3Tx mice may play a role in perseverance of a minimal bacterial numbers thereby maintaining an antigen-dependent stimulation and proliferation.

## INTRODUCTION

A unique animal model, pioneered by a Japanese team, showed that murine autoimmune gastritis (AIG) spontaneously occurring in BALB/c mice subjected to thymectomy (Tx) 3 days after birth (d3Tx mice), predisposes these mice after *Helicobacter pylori* infection, to pathological changes similar to gastric MALT lymphoma (GML) [1] [2] [3]. We recently reproduced in the laboratory this mouse model [4]. A major difference with results from previous studies was the very low incidence of atrophic gastritis or overt inflammation in non-infected d3Tx mice, contrary to the high incidence reported in the previous studies. We postulated that the low incidence of autoimmune gastritis may be related to the infection protected status of our mouse colony and/or differences in diet.

During prenatal and early postnatal life the thymus generates a pool of naive T cells and thus establishes a high degree of diversity in the TCR repertoire, while also playing an important role for the negative selection of potentially self-reactive T cells, which is crucial to avoid the development of autoimmunity [5]. Autoimmune diseases had been shown to develop in some mouse strains when thymectomy is performed at 3 days of age (d3Tx). This could be related to insufficient T cell regulation after thymectomy, possibly by creating a defect in regulatory T (Treg) cells [6]. Thymectomy at 3 days after birth is indeed thought to be followed by a lack of regulatory T cells. It has been proposed that Tregs are exported from the thymus to the periphery after the third day postnatal [7]. This hypothesis is based on the observation that this surgery can lead to the development of organ-specific autoimmune disorders in mice, which are prevented by the early transfer of adult CD25^+^CD4^+^ T cells [7].

In contrast, Monteiro *et al*. found that presence of Treg containing compartment is not affected by neonatal thymectomy and does not lead to a susceptibility to d3Tx-induced autoimmune disease [8]. In fact, they showed that thymectomy-induced gastritis is not caused by the absence of Treg; instead the presence of “gastritogenic” T cell clones is responsible for susceptibility to disease. The expansion of such clones is favored by lymphopenic conditions and, in particular, the reduced Treg/effector T cell ratio that is insufficient to control gastritis development.

In humans, the innate immune response induced by *H. pylori* infection is not able to eliminate the pathogen [9]. An adaptive response follows, with the recruitment of B and T cells to the infection site. Most of these T cells are CD4+ T helper (Th) cells, polarized towards a Th1 phenotype under the influence of surrounding chemokines and cytokines. The Th1 lymphocytes produce proinflammatory cytokines against *H. pylori*, but the pathogen is able to manipulate the immune response and to persist chronically [10]. Tregs recruited to the gastric mucosa during the infection play an important role in this persistence and development of a chronic inflammation via the production of regulatory cytokines such as TGFβ and IL-10 [11]. This hypothesis is based on the fact that *H. pylori* is not able to colonize the gastric mucosa of IL10^−/−^ mice. The immune response is known to contribute to chronic gastritis, leading to the development of more serious diseases in some individuals [12] [13] [11].

It has been previously shown that human gastric MALT lymphoma biopsies are infiltrated by large numbers of Foxp3+ Tregs, which can exhibit a suppressive behaviour toward effector T cells and may be essential for optimal tumor B-cell proliferation [14].

This study utilised complementary molecular and immunohistochemistry approaches to investigate the effect of neonatal thymectomy on the Tregs repertoire using previously obtained samples from d3Tx mice, in order to determine whether thymectomy creates a deficit in this T cell subset thereby allowing the emergence of autoimmune phenomena as a prerequisite for GML. The presence of Tregs at the site of infection was also correlated with inflammatory response and bacterial inoculum.

## RESULTS

### Evaluation of total T cells (CD3ε+) and Tregs (Foxp3+) presence in spleens from non-infected mice

**Figure 1:**
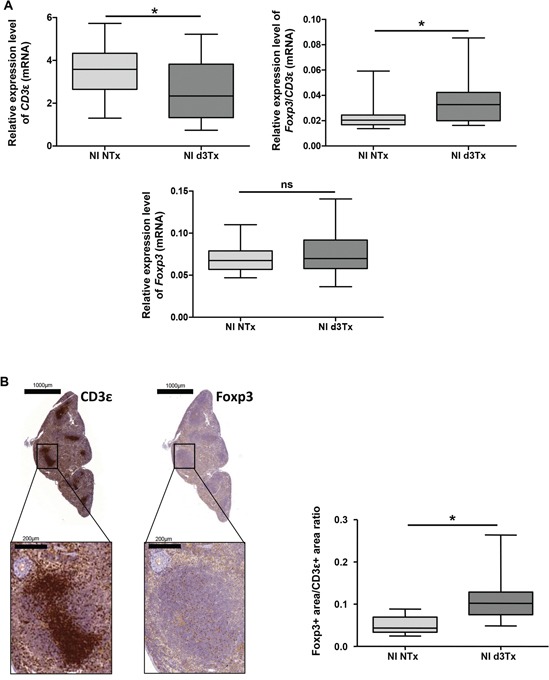
Evaluation of total T cells (CD3ε+) and Tregs (Foxp3+) reserve in non-infected (NI) mice spleens **A.** Relative expression levels of *Foxp3, CD3*ε and *Foxp3/CD3*ε ratio quantified by qRT-PCR in NI non-thymectomised (NTx) (*n* = 10) and NI thymectomised (d3Tx) (*n* = 10) mice spleens. **B.** CD3ε and Foxp3 IHC stainings in one representative spleen from a NI d3Tx mice at 12 months post-infection. The quantification of these stainings, performed as described in the material and methods, on NI NTx (*n* = 10) and NI d3Tx (*n* = 9) mice spleens are represented as a Foxp3/CD3 ratio. Graphic representations are box plots, with the box representing 50% of the values around the median (horizontal line) and the whiskers representing the minimum and maximum of all the data, *p < 0.05, ns = non significant.

Relative expression levels of *Foxp3* and *CD3*ε in non-infected (NI) mice spleens were used to estimate the amount of Tregs within the T cell compartment. The results of qRT-PCR showed a significant deficit of *CD3*ε transcripts in d3Tx mice compared to NTx mice, without a noticeable difference in the quantity of *Foxp3* transcripts. These results suggest that d3Tx mice which were originally selected based on their lymphopenic stage (evaluated 4 weeks post thymectomy) prior to *H. pylori* infection [4], maintained a long-lasting state of lymphopenia. However, when considering relative ratios of *Foxp3/CD*3ε expression levels, a significant enrichment in Tregs within the total T cell compartment was apparent in d3Tx mice spleens in comparison to NTx mice spleens (Figure 1A). A semi-quantitative evaluation of Foxp3 and CD3ε positive cells by immunohistochemistry (IHC) on the corresponding spleens confirmed that Foxp3/CD3ε ratios were higher in d3Tx mice spleens (Figure 1B). In conclusion, GML emergence in d3Tx infected mice is not related to a Tregs deficit.

### Evaluation of the lymphocytic infiltration in infected d3Tx mice stomachs

**Figure 2:**
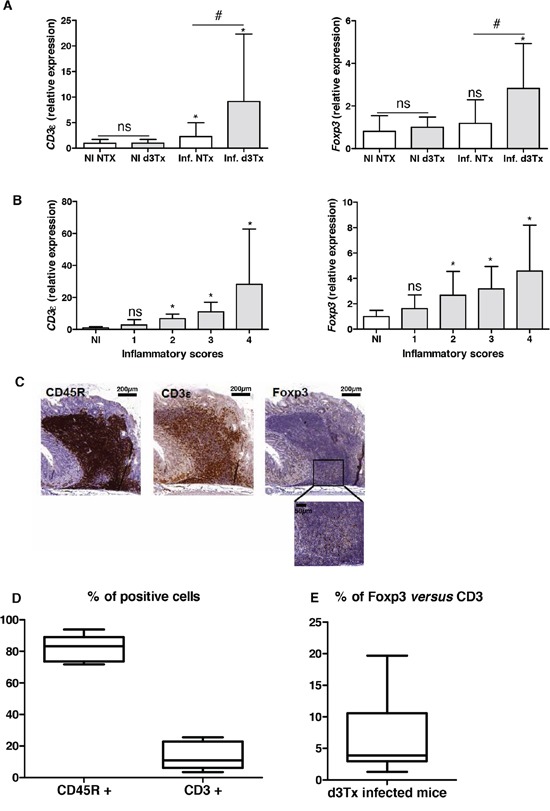
Evaluation of the lymphocytic infiltration in infected d3Tx mice stomachs **A.** Relative expression levels of *Foxp3* and *CD3*ε quantified by qRT-PCR in NI d3Tx (*n* = 10) or NI NTx (*n* = 8) as well as infected d3Tx (*n* = 29) or infected NTx (*n* = 39) mice stomachs. **B.** Evolution of relative expression levels of *Foxp3* and *CD3*ε in d3Tx mice stomachs in comparison with inflammatory scores (NI, *n* = 10) (infected, *n* = 8, 10, 8 and 3 for scores 1, 2, 3 and 4, respectively). In A and B: Data are plotted as bar graphs displaying the mean ± standard deviation. ns: non-significant; *p < 0.05 *versus* NI mice for each group of mice (NTx or d3Tx); #*p* < 0.05 d3Tx compared to NTx. **C.** Example of B cells (CD45R+), T cells (CD3ε+) and Tregs (Foxp3+) after IHC staining on sections of a *H. pylori*-infected d3Tx mouse stomach (strain B47). Scale bars are indicated in μm. A higher magnification of Foxp3 IHC is shown (bar = 50 μm). **D.** Semi-quantitative evaluation of CD45R and CD3ε stainings in leucocyte infiltrates in d3Tx infected mice (*n* = 11). **E.** Semi-quantitative evaluation of Foxp3 and CD3ε stainings in leucocyte infiltrates in d3Tx infected mice (*n* = 11). In D and E, the results are expressed as percentage calculated with the ratio of surface of positive cells/the total surface of selected area for each marker, analyzed with the software Mercator. Graphic representations are box plots, with the box representing 50% of values around the median (horizontal line) and the whiskers representing the minimum and maximum of all the data.

To verify the local Treg infiltration, an evaluation of relative expression levels of *Foxp3* and *CD3*ε was made for NI NTx and d3Tx mice (*n* = 10 for each model) and infected mice (*n* = 39 and *n* = 29, respectively) by qRT-PCR. A significant increase in the amount of *CD3*ε transcripts was found in both types of infected mice, with higher levels evident in d3Tx mice. This correlates with histological inflammatory scores described previously [4]. *Foxp3* relative expression levels were significantly higher in d3Tx infected mice only (Figure 2A). When *Foxp3* and *CD3*ε relative expression levels in d3Tx infected mice were stratified according to the inflammatory scores, they were statistically significant for mice with inflammatory scores higher that 1 (Figure 2B).

Detection of Foxp3, CD3ε and CD45R (B cell surface marker) was carried out by IHC in order to confirm the local presence of Tregs within gastric lymphoid infiltrates in d3Tx infected mice. An example of an IHC staining is presented in Figure 2C and shows a few infiltrating T cells, including a small number of Tregs, in a lymphoid infiltrate made of a majority of B cells in the stomach of an infected d3Tx mouse (Figure 2C). After semi quantification of the CD45R and CD3 IHC stainings in 11 d3Tx infected mice with the highest inflammatory scores (2 to 3 areas with lymphoid infiltrates were considered for each mouse), the proportion of B cells and T cells was around 82.5% (± 8.1%), and 15.6 (± 10.2%) (Figure 2D). Using Ki67 IHC staining, the percentage of proliferating cells among these infiltrates was around 7.6% (± 4.4%) (data not shown). Using the same approach for semi quantification of the CD3ε and Foxp3 IHC stainings in the same infected d3Tx mice, the proportion of Tregs was estimated to be approximately 6.9% of the total T cells (min 1.5, max 19.4%) (Figure 2E). The proportion of Tregs compared to B cells was found to be minimal (around 0.74% ± 0.2%) (data not shown).

In conclusion, Tregs can be detected in gastric GML lesions in d3Tx *H. pylori*-infected mice.

### Estimation of the bacterial load in gastric biopsies from infected mice

**Figure 3:**
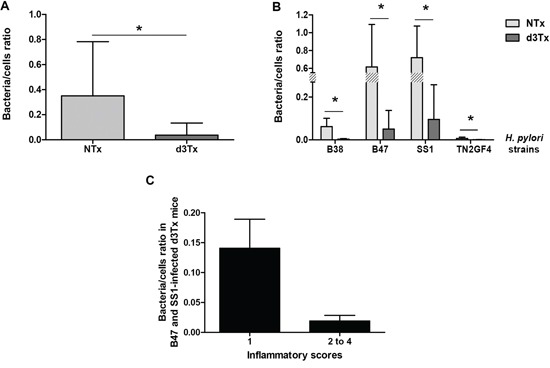
Quantification of the bacterial load in gastric biopsies from infected mice Results obtained by quantitative PCR. **A.** Bacteria/murine cell ratio in NTx (*n* = 40) *versus* d3Tx (*n* = 32) mice stomachs. **B.** Bacteria/murine cell ratio in NTx (*n* = 10 for each bacterial strain) versus d3Tx (*n* = 8 for each bacterial strain) mice stomachs classified according to the infecting bacterial strain: B38, B47, SS1 and TN2GF4. **C.** Bacteria/murine cell ratio in B47- and SS1-infected d3Tx mice stomachs only, classified according to inflammation scores obtained for each mouse (*n* = 3 and 11 for scores of 1 and 2–4, respectively). Data are presented as bar graphs displaying the mean ± standard deviation for each group, *p < 0.05.

The comparison of bacteria-to-cell ratios between NTx and d3Tx mice groups had shown that the number of bacteria per cell was approximately ten times lower in d3Tx mice (average bacteria/cell = 0.037) than in NTx mice (average bacteria/cell = 0.35) (*p < 0.05) (Figure 3A). The same phenomenon was observed when data were separated according to the 4 infecting bacterial strains described previously [4]. For each strain, bacteria/cell ratios were 7 to 20 times lower in d3Tx mice compared to NTx mice. Colonization levels were higher for B47 and SS1 strains in both models, whereas for B38 and TN2GF4 strains, the amount of bacteria per cell in the d3Tx model was close to the method's detection limit (Figure 3B). Interestingly, B47 and SS1 were previously identified as strains with a high potential to induce gastric lymphomagenesis in d3Tx BALB/c mice [4].

For the B47- and SS1-infected d3Tx model, bacteria/cell ratios decreased dramatically for mice in which inflammatory scores were higher than 1 (Figure 3C). At 6 months post-infection, there were no significant differences between the bacterial load in the infected NTx mice compared to NTx mice at 12 month post-infection. The ratio was, however, significantly higher in d3Tx mice (with low inflammation scores) [4] compared to d3Tx at 12 month post-infection at a GML stage (Figure S1). These results confirm the influence of the inflammation on the bacterial persistence.

## DISCUSSION

This study showed that there was no deficit of Tregs in our d3Tx adult mice [4], which is consistent with previous studies conducted on mice 2 to 3 months post-surgery [15] [8]. We confirmed that neonatal thymectomy leads to an enrichment of Tregs within the pool of T cells [15]. These lymphocytes probably multiply from the T cell “library” exported out of the thymus before surgery, despite a poor T cell receptor diversity. Indeed, Monteiro *et al*. showed that neonatal Tregs proliferate extensively after thymectomy during the first week of life and they are maintained throughout adulthood [8].

In the case of infection induced chronic inflammation, Tregs may be involved in pathogen's persistence [13] [16]. On one hand, Tregs are important in protecting the *H. pylori*-infected host against excessive gastric inflammation and disease symptoms; while on the other hand, they may promote bacterial colonization which may increase the risk in *H. pylori*-infected animals to develop GML.

We postulate that Tregs could play a role in the physiopathology in *H. pylori*-induced GML. The presence of Tregs in gastric samples of d3Tx mice developing GML, could be considered as a strategy to protect the gastric mucosa from exaggerated inflammation and tissue damage [17] [16]. It is possible to hypothesise that these Tregs are recruited or induced in the mouse stomachs in order to control inflammation. As shown in a GML murine model involving a *Helicobacter felis* infection, Tregs might be recruited by chemokines such as CCL17 and CCL22 [14], CCL20 and CXCL13 [18]. Concurrent study demonstrated that most of these factors are significantly overexpressed in d3Tx mice developing GML [19]. Interestingly, the administration of anti-CXCL13 antibody has been shown to be promising in patients where the lymphoma does not regress after standard antibiotic treatment [20].

Alternatively, soluble signals derived from tumor B cells, (whose nature is still unknown), may allow the conversion of naive CD4+ T cells into Foxp3+ induced Tregs, similar to that reported for *H. felis* [14]. Indeed, in addition to natural Tregs (nTreg) produced by thymus, peripherally induced Tregs (iTreg) can develop under chronic inflammatory conditions caused by infections or tumours [16]. Unfortunately it was not possible to obtain a suitable commercial antibody for IHC staining which recognizes murine Neuropilin-1, previously described as a suitable marker for distinction between nTregs and iTregs [21].

In a parallel study, neither PCR arrays nor protein arrays [19] showed any overexpression of regulatory cytokines such as IL-10 or TGFβ, typically produced by Tregs. IL-10 is an important cytokine involved in reducing *H. pylori*-induced gastritis and is therefore a particularly important Treg effector at mucosal surfaces. It has also been shown that TGFβ can induce Foxp3 expression in naive T cells and stimulate differentiation into functional iTregs, if present during antigen presentation [16]. The absence of detectable IL-10 and TGFβ transcripts in this study could be explained by the very low level of Treg infiltration in the stomachs in comparison to the amount of CD4+ T cells producing pro-inflammatory cytokines. Previous studies showed that Tregs from d3Tx animals have normal suppressive properties both *in vitro* and *in vivo* [15] [8]. Therefore the local cytokine environment may have important influence. In particular, it has been shown that the presence of Th2 cytokines (which are upregulated in our model) [19] can inhibit the induction of Foxp3 and thus impair the generation of inducible Treg cells [22]. As for the anti-CXCL13 strategy [20], *in vivo* cytokine blocking experiments, for example anti-IL-4 or anti-IL-10, could be evaluated in this model.

The presence of Tregs could also be considered, as already suggested by others [14], as promoters of the B cell proliferation, either by directly stimulating B cells or suppressing T cells. Indeed, it was shown that CD4+ or CD25+ cell depletion *in vivo* induced a regression of the GML in mice, demonstrating that T helper cells are necessary for efficient induction of malignant B cell proliferation *in vitro* bringing into play CD40L in particular and in a suitable cytokine environment [14] as it was identified in our GML mice [19]. However, in our d3Tx mice, the proportion proliferating cells was low and moreover the proportion of Tregs compared to B cells was found to be minimal. Therefore the role of Tregs as promoters of the B cell proliferation may be of less importance in our GML model.

Interestingly, Tregs may also participate in *H. pylori* persistence in chronic inflammation of gastric mucosa. It was previously shown in C57BL/6 mice infected with *Leishmania major* that Tregs inhibited the immune response resulting in the persistence of the pathogen in a dormant state [23]. The results of this study indicate a potential role for Tregs in persistence of a minimal level of colonization allowing the antigenic stimulation process mandatory for B cell proliferation. Indeed, higher Foxp3/CD4 cell ratios and the absolute number of Foxp3 cells in GML were previously found to be significantly higher in *H. pylori* eradication responders compared to non-responders, suggesting that Tregs have a function in regression mechanisms of MALT lymphoma [24] [25].

In summary, although neonatal thymectomy in BALB/c mice may lead to the emergence of GML induced by *H. pylori* infection, it cannot be linked to a global deficit in the Treg reserve. Tregs were detected among lymphoid follicles that form in the gastric mucosa of *H. pylori*-infected d3Tx mice suggesting that they may be induced directly in the stomach mucosa or they may be actively recruited. These cells may act on local T responses being induced in the lymphoid follicles. Local Tregs could also be of major importance for perseverance of a minimal bacterial persistence, thereby maintaining an antigen-dependent disease that could regress after antimicrobial eradication. Tregs depletion by injecting CD25 antibody or the use of DEpletion of REGulatory T cells (DEREG) mice [26] would be useful in the future to assess more precisely the effect on the bacterial load and development of GML in the d3Tx mice.

## MATERIALS AND METHODS

### RNA extraction

RNA from paraffin-embedded spleens of 12-months non-infected (NI) mice were extracted using AllPrep DNA/RNA FFPE Qiagen kit (Qiagen, Courtaboeuf, France): 10 RNAs from NI NTx and d3Tx mice. Gastric RNAs were extracted from gastric biopsies using DNA/RNA/miRNA Universal Qiagen kit (Qiagen): 50 and 40 RNA samples extracted from NTx (10 NI and 40 infected mice) and d3Tx mice (8 NI and 32 infected mice) respectively.

### Pre-amplification of splenic RNA samples

A pre-amplification step of *Foxp3* (forkhead box P3, Treg marker) and *CD3*ε (CD3 epsilon chain, T cell marker) along with a housekeeping gene (HKG) (beta-glucuronidase (*Gusb*)) was carried out with the RT^2^ PreAMP cDNA Synthesis kit (Qiagen). 250 ng of RNA were used for each sample. First, a genomic DNA elimination step was performed, followed by reverse transcription of RNA into complementary DNA (cDNA) according to the manufacturer's instructions. With *Foxp3* (PPM05497F), *CD3*ε (PPM04598A) and the HKG *Gusb* (PPM05490C) primers (RT^2^ qPCR Primer Assay, Qiagen), a PCR was performed in order to amplify these targets, according to the manufacturer's instructions. PCR conditions were as follows: a 95°C DNA denaturation step for 10 minutes followed by 8 amplification cycles of 2 steps, a 95°C denaturation step for 15 seconds and a 60°C primers' hybridization step for 2 minutes. The cDNA samples obtained were then stored at −80°C.

### Gastric RNA sample reverse transcription

The Primescript™ RT Reagent Kit with gDNA Eraser (Perfect Real Time) (Takara, Saint-Germain-en-Laye, France) was used for reverse transcription of all purified RNA samples (750 ng) was performed according to the manufacturer's instructions

### Quantitative real-time (RT)-PCR

Primers used: Primers targeting *Foxp3, CD3*ε and *Gusb* (same as above) were used to assess the relative expression levels of *Foxp3* and *CD3*ε genes in comparison to the HKG *Gusb* in stomachs of both mice groups (NTx and d3Tx) and spleens of NI mice groups.

The amplification conditions were as follows: SYBR^®^ Premix Ex Taq™ Mix (Tli RNaseH Plus) (Takara) was used for all qRT-PCRs. Experiments concerning the expression of *Foxp3, CD3*ε and *Gusb* in spleens and stomachs were carried out in 96-well plates (Bio-Rad, Marnes la Coquette, France) with 1 μl of pure cDNA in a final volume of 25 μl according to the manufacturer's instructions. Each target was tested in triplicate on each sample. PCRs were performed in real-time PCR thermocycler CFX96™ (Bio-Rad) at the TBM-Core real-time PCR platform (University of Bordeaux).

Concerning the relative quantification of the targets' expression, cycle threshold (Ct) values above 35 were considered as non-specific and therefore not considered for the analysis. For each sample, Ct values obtained for each gene of interest were normalised in relation to the average Ct value obtained for each HKG (ΔCt = Ct_gene of interest_ - Ct_HKG)_. The 2^−ΔCt^ value was then calculated, to express the results as relative expression levels of genes of interest.

Relative expression levels of each target gene for the d3Tx mice group were also correlated with the histological scoring previously obtained [4].

### CD45R, Ki67, Foxp3 and CD3ε immunohistochemistry (IHC)

Sections (3 μm thick) from stomachs of infected d3Tx mice and NTx mice and 20 spleens of NI d3Tx and NTx mice (10 in each group) were prepared, put on glass slides and deparaffined then rehydrated. Antigenic sites were uncovered with Tris EDTA (pH 9 for CD45R, Foxp3 and CD3ε IHC or pH6 for Ki67 IHC), 30 min at 100°C and then 30 min at room temperature. Three percent hydrogen peroxide (Sigma-Aldrich, Saint Quentin Fallavier, France) was used to inactivate endogenous peroxidases. Rabbit anti-murine Foxp3 (1/1500, ab54501, Abcam, Paris, France) and goat anti-murine CD3ε (1/1000, M-20 clone, CliniSciences, Nanterre, France) polyclonal primary antibodies, along with rat anti-murine CD45R monoclonal primary antibody (1/100, RA3-6B2 clone, Santa Cruz, CA, USA) were used for detection of Tregs, T and B cells, respectively. Rat anti-murine Ki67 antibody (1/5, clone 16A8, BioLegend, San Diego, CA, USA) was used for detection of proliferating cells. After a hour long incubation, anti-rabbit (EnVision^®^+System-HRP (DAKO, Copenhagen, Denmark)), anti-goat (Immpress Goat Ig (MP 7405, Vector Laboratories, Inc., Burlingame, CA, USA)) and anti-rat (Immpress Rat (MP7444, Vector Laboratories)) secondary antibodies were used to bind primary antibodies previously listed. Liquid DAB+ Substrate Chromogen (DAKO) reagent allowed the revelation of those secondary antibodies. Slides were then counterstained with hemalun (VWR International, Fontenay sous Bois, France), dehydrated and fixed with Eukitt medium (VWR). These slides were then scanned with the MIRAX SCAN (3DHISTECH Ltd, Budapest, Hungary) scanner, and images were read with Panoramic Viewer (3DHISTECH Ltd) software. A semi-quantitative evaluation of the stainings in leucocyte infiltrates was carried out with Mercator (Explora Nova, La Rochelle, France) software. Slide scanning and semi-quantitative evaluation of stainings were performed at the histology platform of our University.

### Quantitative PCR to determine of the bacterial load in gastric biopsies

A quantitative PCR using Fluorescence Resonance Energy Transfer technology targeting DNA coding for *H. pylori* 23S ribosomal RNA (rRNA) previously developed in the laboratory [27] was adapted to our project. Primers described by Oleastro *et al*. were used to amplify the 23S rRNA gene [27] and primers amplifying the *GAPDH* (glyceraldehyde 3-phosphate dehydrogenase) gene were designed for the present study (mGapdh1-F: CTGCAGGTTCTCCACACCTATG; mGapdh1-R: GAA TTTGCCGTGAGTGGAGTC). Every target was tested in duplicate on all samples.

A standard curve was prepared using serial dilutions of a DNA extract from a CFU/mL calibrated bacterial suspension of the *H. pylori* SS1 strain and another from a DNA extract of the m-ICcl2 murine epithelial cell line available in the laboratory. The LightCycler^®^ 480 SYBR^®^ Green I Master Mix (Roche Diagnostics, Basel, Switzerland), compatible with the LightCycler® 480 (Roche Diagnostics) thermocycler was used according to the manufacturer's instructions. The PCR started with a 95°C DNA denaturation step during 10 min, followed by 45 cycles comprised of 3 steps: a 95°C denaturation step for 10 sec, a 60°C primers' hybridization step for 10 sec and a 72°C elongation step for 15 sec. The final results were expressed as a ratio of bacteria/murine cells. The method's detection limit was around 0.001 bacteria/cell.

### Statistical analysis

Statistical analyses were performed with GraphPad Prism 5.01 (GraphPad Software, Inc, La Jolla, CA, USA). Means were compared by the nonparametric Mann-Whitney test. Differences were considered significant when p was inferior to 0.05 (*p < 0.05).

## SUPPLEMENTARY FIGURES AND TABLES


