# Supplementary material for: Regulatory T cells may participate in Helicobacter pylori persistence in gastric MALT lymphoma: lessons from an animal model
Source: Oncotarget. 2015 Dec 7;7(3):3394–402. doi: 10.18632/oncotarget.6492 (PMC4823114; doi:10.18632/oncotarget.6492)
Supplement: Supplementary file 1 [file oncotarget-07-3394-s001.pdf]

SUPPLEMENTARY FIGURE

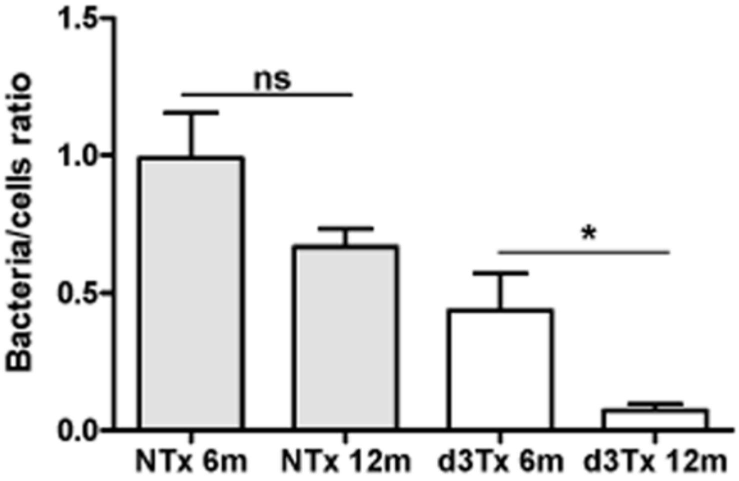

Supplementary Figure S1: Comparison of the bacterial load in gastric biopsies of NTx and d3Tx infected mice at 6 and 12 months post-infection. Results obtained by quantitative PCR. Grey bars represent the bacteria/murine cell ratio in NTx mice at 6 and 12 months post-infection ($n = 38$ and $n = 40$, respectively), white bars for data obtained in d3Tx ($n = 36$ and $n = 32$, respectively). Data are plotted as bar graphs displaying the mean ± standard deviation for each group, $*p < 0.05$.
